# Supplementary material for: A new confuciusornithid bird with a secondary epiphyseal ossification reveals phylogenetic changes in confuciusornithid flight mode
Source: Commun Biol. 2022 Dec 21;5:1398. doi: 10.1038/s42003-022-04316-6 (PMC9772404; doi:10.1038/s42003-022-04316-6)
Supplement: Supplementary file 2 — Supplementary Information [file 42003_2022_4316_MOESM2_ESM.doc]

**A new confuciusornithid bird with a secondary epiphyseal ossification reveals phylogenetic changes in confuciusornithid flight mode**

Renfei Wang^1,2^, Dongyu Hu^2*^, Meisheng Zhang^1^, Shiying Wang^2^, Qi Zhao^3^, Corwin Sullivan^4,5^ & Xing Xu^2,3,6*^

^1^ College of Earth Sciences, Jilin University, Changchun, China

^2^ Shenyang Normal University, Paleontological Museum of Liaoning, Key Laboratory for Evolution of Past Life in Northeast Asia, Liaoning Province, Shenyang, China

^3^ Key Laboratory of Vertebrate Evolution and Human Origins, Institute of Vertebrate Paleontology and Paleoanthropology, Chinese Academy of Sciences, Beijing, China

^4^ Department of Biological Sciences, University of Alberta, Edmonton, AB, Canada

^5^ Philip J. Currie Dinosaur Museum, Wembley, AB T0H 3S0, Canada

^6^ Center for Vertebrate Evolutionary Biology, Yunnan University, Kunming, China

Contents

1. Supplementary Table 1
2. Supplementary character scorings for cladistic analysis
3. Supplementary Table 2-1, 2, 3
4. Supplementary Table 3
5. Supplementary Figure 1
6. Supplementary References

**Supplementary Table 1. Measurements (in mm) of *Confuciusornis shifan*** **(PMoL-AB00178).**

| Element | Length | Element | Length |
| --- | --- | --- | --- |
| Skull | 46.3 | Minor digit phalanges: 1, 2, 3, 4 | --, 9.0, 10.8, 7.5 |
| Pygostyle | 21.7 | Ilium | 23.0 |
| Scapula | 32.3 | Pubis | 42.2 |
| Coracoid | 17.3 | Ischium | 16.4 |
| Humerus | 40.8 | Femur | 33.9 |
| Ulna | 35.7 | Tibiotarsus | 39.8 |
| Radius | 35.1 | Tarsometatarsus | 21.4 |
| Carpometacarpus | 20.4 | Digit I phalanges: 1, 2 | 4.3, 3.7 |
| Alular metacarpal | 7.1 | Digit II phalanges: 1, 2, 3 | 5.9, 6.1, 7.3 |
| Alular digit phalanges: 1, 2 | 15.9, 14.1 | Digit III phalanges: 1, 2, 3, 4 | 6.3, 4.3, 5.4, 5.2 |
| Major digit phalanges: 1, 2, 3 | 13.7, 15.4, 3.2 | Digit IV phalanges: 1, 2, 3, 4, 5 | 3.6, 2.6, 2.5, 4.1, 5.9 |

**Scorings for *Confuciusornis shifan* using the matrix of Wang et al.^1^.**

*Confuciusornis shifan*

?023??13????????0??????0?0???????1?11??0100?100???2??01101?120010???240?0?0?00??01?0?0?000??0?020?010000000?????????????00000000210?0000??0100001002?00??21210101101000000002100200000000010101121000310112??0??1???10000?1?100?0100??021?0200?0011???00003000100001100000??1?000?101010

**Supplementary Table 2-1. Abbreviations for the limb bone measurements given in Supplementary Table 2-2.**

| HL | Length of humerus |
| --- | --- |
| dpL | Length of deltopectoral crest |
| bcL | Length of bicipital crest |
| deHW | Dorsoventral width of distal end of humerus |
| dHW | Dorsoventral width at midshaft of humerus |
| UL | Length of ulna |
| peUW | Dorsoventral width of proximal end of ulna |
| dUW | Craniocaudal width at midshaft of ulna |
| RL | Length of radius |
| CmL | Length of carpometacarpus |
| DCmW | Distance from cranial edge of major metacarpal to the caudal edge of the minor metacarpal, at midshaft of carpometacarpus |
| L_Prim_ | Length of longest primary feather |
| L_Prim_ (e) | Estimated L_Prim_ (used when the primary feathers were not adequatedly preserved) |
| FL | Length of femur |
| dFWcc | Craniocaudal width at midshaft of femur |
| TL | Length of tibiotarsus |
| TmL | Length of tarsometatarsus |

**Supplementary Table 2-2. Measurements of 11 confuciusornithid specimens.**

*E. zhengi* IVPP V 11977: all data from Serrano et al.^2,3^.

*C. dui* IVPP V11521: all data from measuring a high-resolution image.

*Y. confucii* IVPP V18929: HL, UL, RL, CmL, FL, TL and TmL from Wang and Zhou^4^; dpL, bcl, deHW, dHW, peUW, dUW, DCmW and dFWcc from measuring a high-resolution image in Wang and Zhou^4^; L_Prim_(e) estimated as explained in the note accompanying Supplementary Table 2-3.

*Ch. hengdaoziensis* GMV-2129: HL, UL, RL, CmL, FL, TL and TmL from Chiappe et al.^5^; dpL, bcl, deHW, dHW, peUW, dUW, DCmW, dFWcc, and L_Prim_ from measuring a high-resolution image in Chiappe et al.^5^.

*C. sanctus* IVPP V 11640, IVPP V 11374, and MCFO-0374: all data from Serrano et al.^2,3^.

*C. sanctus* IVPP 11372 and IVPP 11375: all data except L_Prim_ from Serrano et al.^2,3^ ; L_Prim_ from measuring a high-resolution image.

*C.* sp. IVPP 11370: all data except L_Prim_ from Serrano et al.^2,3^; L_Prim_(e) estimated as explained in the note accompanying Supplementary Table 2-3.

*C. shifan* PMoL-AB00178: all data except L_Prim_ from directly measuring the specimen with digital calipers; L_Prim_(e) estimated as explained in the note accompanying Supplementary Table 2-3.

PMoL, Paleontological Museum of Liaoning, Shenyang;

IVPP, Institute of Vertebrate Paleontology and Paleoanthropology, Beijing;

GMV, National Geological Museum of China, Beijing.

MCFO, CosmoCaixa, Barcelona, Barcelona.

| Measurement  (mm) | *E. zhengi* | *C. dui* | *Y. confucii* | *Ch. hengdaoziensis* | *C.* sp | *C. sanctus* |  |  |  |  | *C. shifan* |
| --- | --- | --- | --- | --- | --- | --- | --- | --- | --- | --- | --- |
|  | IVPP V11977 | IVPP V11521 | IVPP V18929 | GMV-2129 | IVPP V11370 | IVPP  V11640 | MCFO-0374 | IVPP V11372 | IVPP V11375 | IVPP V11374 | PMoL  AB00178 |
| HL | 40.29 | 42.00 | 55.38 | 33.53 | 73.93 | 64.61 | 54.29 | 52.58 | 53.28 | 53.32 | 40.75 |
| dpL | 16.52 | 18.09 | 26.53 | 14.52 | 32.89 | 27.70 | 21.37 | 22.13 | 20.97 | 21.20 | 18.59 |
| bcL | 6.96 | 6.34 | 10.87 | 5.32 | 13.97 | 13.02 | 16.05 | 8.40 | 10.73 | 11.14 | 8.23 |
| deHW | 5.49 | 6.72 | 10.43 | 7.09 | 14.34 | 12.04 | 10.09 | 11.29 | 11.36 | 11.96 | 9.10 |
| dHW | 3.16 | 3.61 | 6.34 | 3.04 | 7.64 | 6.14 | 5.19 | 5.31 | 5.21 | 5.92 | 3.66 |
| UL | 35.66 | 39.00 | 52.25 | 31.97 | 58.12 | 54.79 | 44.67 | 47.23 | 44.66 | 46.08 | 35.73 |
| peUW | 5.17 | 4.91 | 7.50 | 4.00 | 9.30 | 7.88 | 6.84 | 5.95 | 6.98 | 7.63 | 4.57 |
| dUW | 3.21 | 3.17 | 5.01 | 2.84 | 5.11 | 5.11 | 3.86 | 4.06 | 4.08 | 4.14 | 3.20 |
| RL | 33.23 | 38.16 | 50.44 | 31.07 | 55.69 | 54.85 | 42.64 | 44.96 | 43.01 | 43.40 | 35.12 |
| CmL | 20.21 | 19.00 | 28.75 | 17.07 | 34.65 | 32.75 | 26.93 | 26.80 | 25.45 | 25.51 | 20.42 |
| DCmW | 4.19 | 3.91 | 6.62 | 4.02 | 8.09 | 7.39 | 5.70 | 5.30 | 7.10 | 6.49 | 4.93 |
| L_Prim_ | 119.67 | 170.00 |  | 110.00 |  | 205.12 | 168.66 | 185.00 | 180.00 | 175.39 |  |
| L_Prim_(e) |  |  | 189.66 |  | 231.82 |  |  |  |  |  | 134.75 |
| FL | 35.79 | 36.00 | 40.06 | 33.46 | 59.96 | 54.61 | 43.63 | 45.14 | 45.86 | 46.53 | 33.92 |
| dFWcc | 3.57 | 3.83 | 4.68 | 3.71 | 6.48 | 6.27 | 5.53 | 4.60 | 5.07 | 5.57 | 3.76 |
| TL | 42.10 | 41.00 | 50.32 | 36.59 | 69.93 | 64.22 | 52.09 | 51.95 | 53.55 | 53.78 | 39.75 |
| TmL | 23.06 | 19.50 | 23.17 | 20.64 | 32.65 | 30.85 | 25.07 | 25.25 | 26.02 | 26.82 | 21.35 |

**Supplementary Table 2-3. Measured and estimated longest primary feather lengths for confuciusornithid specimens.**

|  | *E. zhengi* | | | *C. dui* | | *Y. confucii* | | | | | | *Ch.*  *hengdaoziensis* | | | | *C.* sp | | *C. sanctus* | | | | |  | | | |  | | |  | |  | | *C. shifan* | | | |
| --- | --- | --- | --- | --- | --- | --- | --- | --- | --- | --- | --- | --- | --- | --- | --- | --- | --- | --- | --- | --- | --- | --- | --- | --- | --- | --- | --- | --- | --- | --- | --- | --- | --- | --- | --- | --- | --- |
|  | | IVPP  V11977 | | IVPP V11521 | | | IVPP  V18929 | | | | | | GMV-2129 | | | IVPP  V11370 | | | IVPP  V11640 | | | MCFO  -0374 | | | IVPP  V11372 | | | IVPP  V11375 | | | IVPP  V11374 | | | | PMoL  AB00178 | | |
| L_Prim_ (mm) | 119.67 | | 170.00 | |  | | | | | | 110.00 | | | |  | | 205.12 | | | 168.66 | | | | 185.00 | | 180.00 | | | 175.39 | | | |  | | | |  |
| L_Prim_(e) (mm) | 133.76 | | 139.10 | | 189.70 | | | | | 114.85 | | | | | 231.88 | | 211.64 | | | 175.11 | | | | 176.11 | | 171.64 | | | 173.75 | | | | 134.79 | | | |  |
| HL+UL+CmL (mm) | 96.16 | | 100.00 | | 136.38 | | | | | 82.57 | | | | | 166.70 | | 152.15 | | | 125.89 | | | | 126.61 | | 123.39 | | | 124.91 | | | | 96.90 | | | |  |
| L_Prim_ */* (HL+UL+CmL) | 1.244 | | 1.700 | |  | | | | | | 1.332 | | | |  | | 1.348 | | | 1.340 | | | | 1.461 | | 1.459 | | | 1.404 | | | |  | | | |  |
|  | |  | | | | | |  |  | | | | |  | | |  | | | |  | | | |  | |  | | |  | |  | | | |  | |

Note: We calculated L_Prim_(e) values according to the empirically derived equation L_Prim_(e) = 1.391 * (HL + UL + CmL). The coefficient 1.391 represents the average value of L_Prim_ */* (HL+UL+CmL) for the eight specimens in which L_Prim_ could be measured, excluding both the highest value (1.700, for *C. dui*) and the lowest value (1.244, for *E. zhengi*). The L_Prim_(e) given by our equation is within 5% of L_Prim_ in most specimens in which the latter can be measured, the only exceptions being *C. dui* and *E. zhengi*, so we are confident that the equation provides reasonable estimates of the lengths of the longest primary feathers of *Y. confucii*, *C. shifan* and *C*. sp. IVPP V11370.

**Supplementary Table 3. Estimated body mass (BM), wingspan (B), lift surface area (S_L_), wing loading (WL) and aspect ratio (AR) for 11 confuciusornithid specimens.**

|  | | BM (g) | | B (mm) | | S_L_ (cm^2^) | WL (g/cm^2^) | AR | |
| --- | --- | --- | --- | --- | --- | --- | --- | --- | --- |
| *E. zhengi*  IVPP V 11977 | | 206.5 ± 34.9 | | 488.66 ± 12.71 | | 264.57 ± 23.28 | 0.780 ± 0.15 | 9.02 ± 0.96 | |
| *C. dui*  IVPP V 11521 | | 217.34 ± 36.72 | | 575.94 ± 14.97 | | 407.36 ± 35.85 | 0.533 ± 0.10 | 8.14 ± 0.86 | |
| *Y. confucii*  IVPP V 18929 | | 508.00 ± 85.85 | | 723.00 ± 18.80 | | 766.06 ± 67.41 | 0.663 ± 0.13 | 6.82 ± 0.72 | |
| *Ch. hengdaoziensis*  GMV-2129 | | 138.19 ± 23.35 | | 430.25 ± 11.19 | | 328.87 ± 28.94 | 0.420 ± 0.08 | 5.63 ± 0.60 | |
| *C.* sp  IVPP V 11370 | | 801.28 ± 135.42 | | 891.37 ± 23.18 | | 1233.14 ± 108.52 | 0.650 ± 0.13 | 6.44 ± 0.68 | |
| *C. sanctus*  IVPP V 11640 | | 723.30 ± 122.24 | | 802.43 ± 20.86 | | 957.48 ± 84.26 | 0.755 ± 0.15 | 6.72 ± 0.71 | |
| MCFO-0374 | | 522.39 ± 88.28 | | 664.25 ± 17.27 | | 658.96 ± 57.99 | 0.793 ± 0.15 | 6.69 ± 0.71 | |
| IVPPV 11372 | | 352.24 ± 59.53 | | 689.87 ± 17.94 | | 792.80 ± 69.77 | 0.444 ± 0.09 | 6.00 ± 0.64 | |
| IVPP V 11375 | | 406.93 ± 68.77 | | 673.16 ± 17.50 | | 775.94 ± 68.28 | 0.524 ± 0.10 | 5.84 ± 0.62 | |
| IVPP V 11374 | | 493.58 ± 83.42 | | 667.43 ± 17.35 | | 814.17 ± 71.65 | 0.606 ± 0.12 | 5.47 ± 0.58 | |
| *C. shifan*  PMoL-AB00178 | | 174.53 ± 29.50 | | 518.38 ± 1 3.48 | | 495.68 ± 43.62 | 0.352 ± 0.07 | 5.42 ± 0.57 | |
|  |  | |  | |  | |  | |  |

Note: BM was estimated with the “CONF” equation in Table 4 of Serrano et al.^2^, B with the “MR-B1” equation in Table 6 of Serrano et al.^3^ and S_L_ with the “MR-S_L_ Jeho1=Conf=Ornph2” equation in Table 9 of Serrano et al.^3^. The respective average percent deviations (|%MPE|) between observed values of BM, B, S_L_, WL, and AR, and estimates of these parameters generated by the same equations used in our analysis, were found in previous studies^2,3^ to be 16.9, 2.6, 8.8, 19.4, and 10.6, across large samples of modern birds. These |%MPE| values are adopted here as error terms, giving prediction intervals of the following form: estimated value ± |%MPE| value.

**
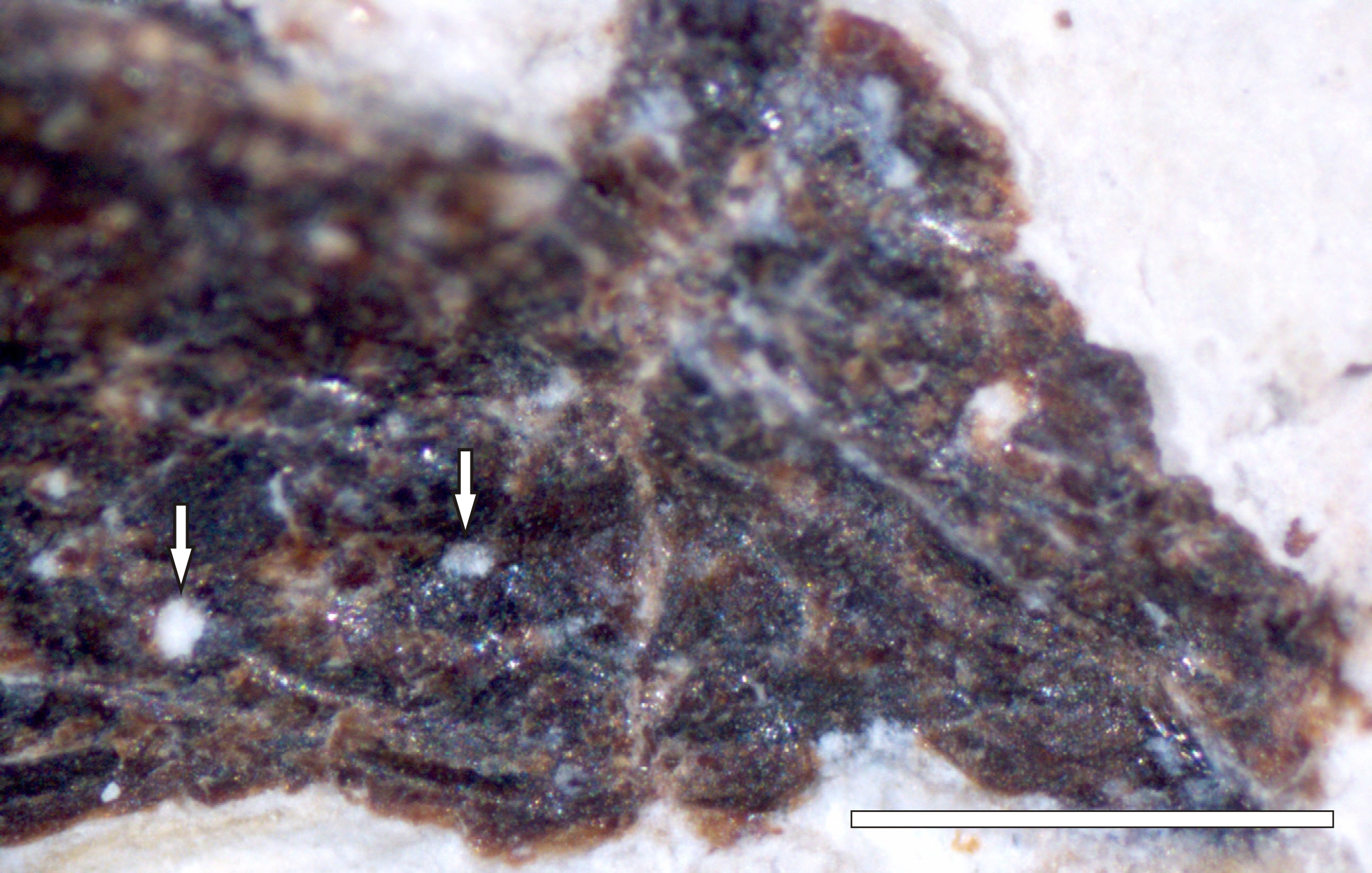
**

**Supplementary Figure 1. Photograph of the distal part of the pygostyle of *Confuciusornis shifan* holotype (PMoL-AB00178).**

Arrows indicate the positions of the distalmost two foramen along the pygostyle. Scale bars: 1 mm.

**Supplementary References**

- - - 1. Wang, M., Lloyd, G. T., Zhang, C. & Zhou, Z. H. The patterns and modes of the evolution of disparity in Mesozoic birds. *Proc. R. Soc*. *B* **288**, 20203105 (2021).
      2. Serrano, F. J., Palmqvist, P., Sanz, J. L. Multivariate analysis of neognath skeletal measurements: implications for body mass estimation in Mesozoic birds. *Zool. J. Linn. Soc.* **173**, 929–955 (2015).
      3. Serrano, F. J., Palmqvist, P., Chiappe, L. M. & Sanz, J. L. Inferring flight parameters of mesozoic avians through multivariate analyses of forelimb elements in their living relatives. *Paleobiology* **43**, 144–169 (2017).
      4. Wang, M. & Zhou, Z. H. A new confuciusornithid (Aves: Pygostylia) from the Early Cretaceous increases the morphological disparity of the Confuciusornithidae. *Zool. J. Linn. Soc.* **185**, 417–430 (2019).
      5. Chiappe, L. M., Ji, S. A., Ji, Q. & Norell, M. A. Anatomy and systematics of the Confuciusornithidae (Theropoda: Aves) from the Late Mesozoic of northeastern China. *Bull. Am. Mus. Nat. Hist*. **242**, 1–89 (1999).
