# Supplementary material for: A new confuciusornithid bird with a secondary epiphyseal ossification reveals phylogenetic changes in confuciusornithid flight mode
Source: Commun Biol. 2022 Dec 21;5:1398. doi: 10.1038/s42003-022-04316-6 (PMC9772404; doi:10.1038/s42003-022-04316-6)
Supplement: Supplementary file 3 — Reporting Summary [file 42003_2022_4316_MOESM3_ESM.pdf]

nature portfolio

Corresponding author(s): Dongyu Hu

Last updated by author(s): Nov 15, 2022

Reporting Summary

Nature Portfolio wishes to improve the reproducibility of the work that we publish. This form provides structure for consistency and transparency in reporting. For further information on Nature Portfolio policies, see our Editorial Policies and the Editorial Policy Checklist.

Statistics

For all statistical analyses, confirm that the following items are present in the figure legend, table legend, main text, or Methods section.

n/a	Confirmed	
☐	☒	The exact sample size (*n*) for each experimental group/condition, given as a discrete number and unit of measurement
☐	☒	A statement on whether measurements were taken from distinct samples or whether the same sample was measured repeatedly
☒	☐	The statistical test(s) used AND whether they are one- or two-sided<br>*Only common tests should be described solely by name; describe more complex techniques in the Methods section.*
☒	☐	A description of all covariates tested
☒	☐	A description of any assumptions or corrections, such as tests of normality and adjustment for multiple comparisons
☒	☐	A full description of the statistical parameters including central tendency (e.g. means) or other basic estimates (e.g. regression coefficient) AND variation (e.g. standard deviation) or associated estimates of uncertainty (e.g. confidence intervals)
☒	☐	For null hypothesis testing, the test statistic (e.g. *F*, *t*, *r*) with confidence intervals, effect sizes, degrees of freedom and *P* value noted<br>*Give P values as exact values whenever suitable.*
☒	☐	For Bayesian analysis, information on the choice of priors and Markov chain Monte Carlo settings
☒	☐	For hierarchical and complex designs, identification of the appropriate level for tests and full reporting of outcomes
☒	☐	Estimates of effect sizes (e.g. Cohen's *d*, Pearson's *r*), indicating how they were calculated

Our web collection on statistics for biologists contains articles on many of the points above.

Software and code

Policy information about availability of computer code

Data collection	No Software was used.
Data analysis	A publicly available software TNT was used to the phylogenetic analysis.

For manuscripts utilizing custom algorithms or software that are central to the research but not yet described in published literature, software must be made available to editors and reviewers. We strongly encourage code deposition in a community repository (e.g. GitHub). See the Nature Portfolio guidelines for submitting code & software for further information.

Data

Policy information about availability of data

All manuscripts must include a data availability statement. This statement should provide the following information, where applicable:

- Accession codes, unique identifiers, or web links for publicly available datasets
- A description of any restrictions on data availability
- For clinical datasets or third party data, please ensure that the statement adheres to our policy

All data generated or analysed during this study are included in this published article (and its supplementary information files).

Human research participants

Policy information about studies involving human research participants and Sex and Gender in Research.

Reporting on sex and gender	*Use the terms sex (biological attribute) and gender (shaped by social and cultural circumstances) carefully in order to avoid confusing both terms. Indicate if findings apply to only one sex or gender; describe whether sex and gender were considered in study design whether sex and/or gender was determined based on self-reporting or assigned and methods used. Provide in the source data disaggregated sex and gender data where this information has been collected, and consent has been obtained for sharing of individual-level data; provide overall numbers in this Reporting Summary. Please state if this information has not been collected. Report sex- and gender-based analyses where performed, justify reasons for lack of sex- and gender-based analysis.*
Population characteristics	*Describe the covariate-relevant population characteristics of the human research participants (e.g. age, genotypic information, past and current diagnosis and treatment categories). If you filled out the behavioural & social sciences study design questions and have nothing to add here, write "See above."*
Recruitment	*Describe how participants were recruited. Outline any potential self-selection bias or other biases that may be present and how these are likely to impact results.*
Ethics oversight	*Identify the organization(s) that approved the study protocol.*

Note that full information on the approval of the study protocol must also be provided in the manuscript.

Field-specific reporting

Please select the one below that is the best fit for your research. If you are not sure, read the appropriate sections before making your selection.

☐ Life sciences ☐ Behavioural & social sciences ☒ Ecological, evolutionary & environmental sciences

For a reference copy of the document with all sections, see nature.com/documents/nr-reporting-summary-flat.pdf

Ecological, evolutionary & environmental sciences study design

All studies must disclose on these points even when the disclosure is negative.

Study description	A new confuciusornithid species from the Lower Cretaceous of western Liaoning of China is reported in this study. Compared to other confuciusornithids, this new species and the recently reported Yangavis confucii both show evidence of stronger flight capability, although the wings of the two taxa differ from one another in many respects. Our aerodynamic analyses under phylogeny indicate that varying modes of flight adaptation emerged across the diversity of confuciusornithids, and to a lesser degree over the course of their ontogeny, and specifically suggest that both a trend towards improved flight capability and a change in flight strategy occurred in confuciusornithid evolution. The new confuciusornithid differs most saliently from other Mesozoic birds in having an extra cushion-like bone in the first digit of the wing, a highly unusual feature that may have helped to meet the functional demands of flight at a stage when skeletal growth was still incomplete. The new find strikingly exemplifies the morphological, developmental and functional diversity of the first beaked birds.
Research sample	Confuciusornithidae is a clade of Early Cretaceous pygostylian birds known from the Jehol Biota of East Asia, representing the earliest known toothless, beaked birds. The new confuciusornithid specimen in this study is from the Jiufotang Formation of the Jehol Biota. It differs from other known confuciusornithids in a number of morphological and developmental features, which have implications for understanding confuciusornithid taxic diversity, morphological and developmental disparity, and flight behavior.
Sampling strategy	In this study, the flight capabilities and strategies of confuciusornithid birds were analyzed, based on the methods of Seranno et al (2015, 2017). The 11 confuciusornthid specimens were used in this analysis. This sample size satisfies the method requirements.
Data collection	The anatomical data in this study were obtained by measuring the specimens directly or high-resolution images published in the literatures, or from estimated measurements based on the methods of Serrano et al. 2018 (see Supplementary table S2 for details).
Timing and spatial scale	The anatomical data in this study were collected between 2018 and 2020.
Data exclusions	No data were excluded from the analyses.
Reproducibility	In this study, the phylogenetic analysis and several flight parameters were repeatedly calculated several times separately in TNT v1.5 and by the methods of Seranno et al. (2015, 2017), and the same results were achieved.
Randomization	The anatomical data of all published and completely preserved specimens were measured.
Blinding	All published and completely preserved specimens were chosen for collecting the anatomical data.

Did the study involve field work? ☐ Yes ☒ No

Reporting for specific materials, systems and methods

We require information from authors about some types of materials, experimental systems and methods used in many studies. Here, indicate whether each material, system or method listed is relevant to your study. If you are not sure if a list item applies to your research, read the appropriate section before selecting a response.

Materials & experimental systems

n/a	Involved in the study
☒	☐ Antibodies
☒	☐ Eukaryotic cell lines
☐	☒ Palaeontology and archaeology
☒	☐ Animals and other organisms
☒	☐ Clinical data
☒	☐ Dual use research of concern

Methods

n/a	Involved in the study
☒	☐ ChIP-seq
☒	☐ Flow cytometry
☒	☐ MRI-based neuroimaging

Palaeontology and Archaeology

Specimen provenance	The specimen PMoL-AB00178 is from a Lower Cretaceous strutum in Jianchang County of Liaoning Province, China.
Specimen deposition	The specimen is deposited at the Paleontological Museum of Liaoning (PMoL-AB00178) to permit free access by other researchers.
Dating methods	No new dates are provided.

☒ Tick this box to confirm that the raw and calibrated dates are available in the paper or in Supplementary Information.

Ethics oversight	The Paleontological Museum of Liaoning has approved the study protocol.

Note that full information on the approval of the study protocol must also be provided in the manuscript.
